# 583 Photos of Burn Wounds Can Help Reduce Over-Triage and Prevent Unnecessary Ambulance Transfer

**DOI:** 10.1093/jbcr/irac012.211

**Published:** 2022-03-23

**Authors:** Jacita A McCourt, Eli Strait, Jeanne Lee

**Affiliations:** UC San Diego Health Regional Burn Center, San Diego, California; UC San Diego Health Regional Burn Center, San Diego, California; UC San Diego Health Regional Burn Center, San Diego, California

## Abstract

**Introduction:**

Burn wounds can be difficult to assess for providers outside the burn center and can result in over triage. The combination of photos of burn wounds with a clinical history can help burn practitioners make appropriate triage decisions, including immediate ambulance transfer vs scheduling an outpatient follow up appointment. Appropriate photo triage can help reduce healthcare costs by eliminating both unnecessary transfers to the burn center and overburdening burn resources. This performance improvement project involved the development of a secure photo sharing web portal and photo triage clinical pathway to help burn practitioners appropriately triage burn patients being evaluated at health care facilities within the catchment area of an American Burn Association verified adult and pediatric burn center.

**Methods:**

Existing technology was used to develop a burn photo sharing web portal that can be easily accessed by providers outside the burn center. A new clinical pathway for burn photo triage was developed. Education was formulated for nurses and providers within the burn center and for referring facilities. Retrospective data was collected for the 4 years of ambulance transfers captured in the outpatient burn registry prior to the implementation of the photo triage clinical pathway. Comparison data was also abstracted for the first year after implementation. Patients were categorized as over triaged or appropriately triage based on the first set of photos captured in the EMR.

**Results:**

In the pre-triage years there were a total of 242 ambulance transfers to the outpatient burn clinic. 150 (62%) of those patients were appropriately triaged, while 92 (38%) were over triaged. In the year following implementation there were 27 ambulance transfers to the outpatient burn clinic. 25 (92.6%) of these patients were appropriately triaged while 2 (7.4%) were over triaged. Overall ambulance transfers to the outpatient burn clinic dropped by more than 50% (average of 60.5 transfer per year down to 27 after implementation).

**Conclusions:**

Patients with burn injuries at referring facilities were more appropriately triaged when using photos of wounds which ultimately reduced the number of unnecessary ambulance transfers.

